# Longitudinal investigation of nasopharyngeal pneumococcal carriage in early childhood: The PATCH birth cohort study

**DOI:** 10.1371/journal.pone.0237871

**Published:** 2020-08-20

**Authors:** Ming-Han Tsai, Sui-Ling Liao, Chih-Yung Chiu, Hsiang-Ju Shih, Man-Chin Hua, Tsung-Chieh Yao, Shen-Hao Lai, Kuo-Wei Yeh, Li-Chen Chen, Yi-Jung Chang, Jing-Long Huang

**Affiliations:** 1 Department of Pediatrics, Chang Gung Memorial Hospital, Keelung, Taiwan; 2 Chang Gung University College of Medicine, Taoyuan, Taiwan; 3 Molecular Infectious Disease Research Center, Chang Gung Memorial Hospital, Taoyuan, Taiwan; 4 Division of Pulmonology, Department of Pediatrics, Chang Gung Children’s Hospital, Taoyuan, Taiwan; 5 Division of Allergy, Asthma, and Rheumatology, Department of Pediatrics, Chang Gung Children’s Hospital, Taoyuan, Taiwan; 6 Department of Pediatrics, New Taipei Municipal TuCheng Hospital, Chang Gung Memorial Hospital and Chang Gung University, New Taipei, Taiwan; 7 Department of Pediatrics, Chang Gung Children’s Hospital, Taoyuan, Taiwan; Hong Kong Children's Hospital, HONG KONG

## Abstract

*Streptococcus pneumoniae* is a common cause of infectious diseases such as pneumonia and sepsis. Its colonization is thought to be the first step in the development of invasive pneumococcal diseases. This study aimed to investigate pneumococcal colonization patterns in early childhood. A longitudinal birth cohort study was conducted for investigating nasopharyngeal colonized pneumococci at 1, 6, 12, 18, 24, and 36 months of age, particularly focusing on the serotype distribution and antimicrobial susceptibilities. Pneumococcal conjugate vaccine (PCV) effect on nasopharyngeal colonization was also assessed. During 2013–2017, 855 infants were enrolled and a total of 107 isolates were recovered from 95 infants during the first three years of life. In this period, the prevalence of pneumococcal colonization increased, with values ranging from 0.2% (2/834) at 1 month of age to 5.9% (19/323) at 36 months of age. The investigation of serotype revealed that 81.1% (73/90) belonged to the non-PCV13 serotypes–23A, 15A, 15C, and 15B. Moreover, PCV13 serotypes significantly decreased during 2014–2015, when routine PCV13 vaccination was initiated in Taiwan. PCV13 introduction may lead to the reduction in the rates of pneumococcal isolates resistant (R) to penicillin. Under conditional PCV13 vaccination, pneumococcal isolates primarily belonged to non-PCV13 serotypes. This non-PCV13 serotype replacement exhibited lower rates of penicillin R isolates, suggesting that PCV13 administration may reduce the antibiotic-nonsusceptible pneumococcal disease burden and antibiotic use.

## Introduction

*Streptococcus pneumoniae* is one of the main causes of infectious diseases, including pneumonia, meningitis, and sepsis, which are associated with high morbidity and mortality [1,2]. It is estimated that *S*. *pneumoniae* is responsible for 1.6 million deaths annually, particularly affecting young children and elderly persons [3]. *S*. *pneumoniae* is commonly found in the nasopharyngeal flora of healthy persons; pneumococcal colonization is thought to be the first step in the development of invasive diseases [4,5]. In addition, the widespread use of antimicrobial agents has led to the high prevalence of antimicrobial-resistant pneumococci, which has made treatment of pneumococcal infections challenging [2]. Therefore, vaccines have been developed to prevent pneumococcal infections.

In 2000, pneumococcal conjugate vaccine (PCV) was globally introduced to combat the invasive pneumococcal disease in young children [6]. The first PCV was the 7-valent PCV, which contained serotypes 4, 6B, 9V, 14, 18C, 19F, and 23F. Subsequently, the vaccine was expanded to cover ten (PCV7 and serotypes 1, 5, and 7F) and thirteen (PCV10 and serotypes 3, 6A, and 19A) serotypes [7]. In Taiwan, PCV7 was introduced for private use in 2005 [8,9]. Nonetheless, an increasing number of invasive infections occurred due to the non-vaccine serotype 19A [10]. Therefore, PCV10 was introduced in Taiwan in 2010, with minimal or no notable improvement, possibly because these three serotypes (serotypes 1, 5, and 7F) exhibited a low occurrence in Taiwan [11]. In 2011, PCV13 was introduced for private use, as an initial step. A national catch-up PCV13 program was launched in March 2013 for children aged 2–5 years and was then expanded, from January 2014 to December 2014, to also cover children aged 1–5 years [12]. By the end of 2014, the age-appropriate immunization rate reached 80%, according to the report from the Center for Disease Control in Taiwan [2]. PCV13 was not introduced as a routine immunization for all infants until January 2015 [12].

A longitudinal birth-cohort study was conducted to improve the understanding of pneumococcal colonization in children in the context of high antibiotic selection pressure and conditional use of pneumococcal vaccination in Taiwan. Infants were prospectively examined for nasopharyngeal colonized bacteria at specified time points. Both the distribution of serotypes and the evolution of pneumococcal antibiotic susceptibility were assessed.

## Methods

### Study population

The Prediction of Allergen in Taiwanese Children (PATCH) Study, an ongoing prospective study launched in 2013 following a birth cohort of infants, was designed to investigate the bacterial colonization and the factors related to the development of asthma and other allergic diseases. Women in the third trimester of pregnancy, admitted at the Obstetrics Clinic of Keelung Chang Gung Memorial Hospital, were invited to participate in the study. Infants exhibiting severe congenital abnormalities, low birth weight (<2000 g), or requiring mechanical ventilation at any point since birth were excluded. The institutional review boards of Keelung Chang Gung Memorial Hospital approved the study project and a written informed consent was obtained from the mother of each participant.

### Sample and clinical parameter collection

Mothers were requested to bring the enrolled infants (1, 6, 12, 18, 24 and 36 months of age) to the Pediatric Clinic of Keelung Chang Gung Memorial Hospital for *S*. *pneumoniae* detection from nasopharyngeal swabs. One swab per infant per visit study was taken. Questionnaire surveys were conducted at each visit to obtain information regarding demographic data, housing and living conditions, socioeconomic status, risk factors for colonization, history of respiratory tract infection and pneumococcal vaccination, and other clinical parameters.

Nasopharyngeal swabs were collected with separate cotton-tipped swabs (Copan Swab Applicator, Copan Diagnostics Inc., Brescia, Italy), through the nose, into the nasopharyngeal spaces at the scheduled visits. After placed into a transported medium, swabs were transported to the microbiology laboratory within 2 h after collection and cultured for bacteria by using standard methods for identification [13]. All swabs were plated within 6 hours of sampling on blood agar plates with 5% sheep’s blood to isolate *S*. *pneumoniae*.

### Antimicrobial susceptibility testing

Pneumococcal isolates were examined for antimicrobial susceptibility to penicillin, levofloxacin, ceftriaxone, and trimethoprim-sulfamethoxazole using the E-test method (bioMerieux, Durham, USA). Susceptibility and resistance were defined in accordance with the criteria suggested by the Clinical and Laboratory Standards Institute (CLSI) [14]. The minimum inhibitory concentrations (MICs) used were based on the criteria recommended by the CLSI for meningeal and non-meningeal infections. For consistency of data interpretation in the study, isolates were categorized as susceptible (S; penicillin, ≤ 0.06 mg/L; ceftriaxone, ≤ 0.5 mg/L), intermediate (I; penicillin, 0.12–1 mg/L; ceftriaxone, 1 mg/L), and resistant (R; penicillin, > 2 mg/L; ceftriaxone, > 1 mg/L). According to the CLSI criteria, although susceptibility in the I category indicated that *β*-lactam remained effective for non-meningeal infections, the result should be interpreted as resistant for meningeal infections.

### Serotyping of pneumococci and multi-locus sequence typing

Serotypes of pneumococcal isolates were determined using commercialized antisera (Statens Serum Institut, Copenhagen, Denmark) and polymerase chain reaction (PCR) methods [15,16]. Multi-locus sequence typing (MLST) was performed by PCR-sequencing of a set of pneumococcal housekeeping genes, including *ardE*, *gdh*, *gki*, *recP*, *spi*, *xpt*, and *ddl* [17]. The sequence data were compared with the MLST database (http://pubmlst.org/spneumoniae/). New alleles and allelic profiles were submitted to the database curator for assignment of the sequence type (ST) numbers. eBURST analysis was utilized to group STs into clonal complexes (STs shared six of the seven MLST loci).

### Statistical analysis

Data were analyzed using SPSS, version 12.0 (SPSS inc., Chicago, IL). Student’s *t*-test was used to analyze numerical data. If the data were not normally distributed, the Mann-Whitney *U*-test was used for analysis. Chi-square test or Fisher’s exact test, when appropriate, was used to analyze categorical data. In addition, the linear trend of the proportion of isolates with PCV13 coverage and rates of penicillin R isolates across the study period were tested using the Cochran–Armitage chi-square test. To investigate factors associated with pneumococcal carriage by univariate analysis, each colonized infant was frequency-matched with the non-colonized infants on variables of year of study, season, age and gender. Variables for which *P*<0.1 were chosen for model selection of multivariate analysis. *P*<0.05 was considered statistically significant.

## Results

### Demographics and factors associated with pneumococcal colonization among the study population

**Fig 1:**
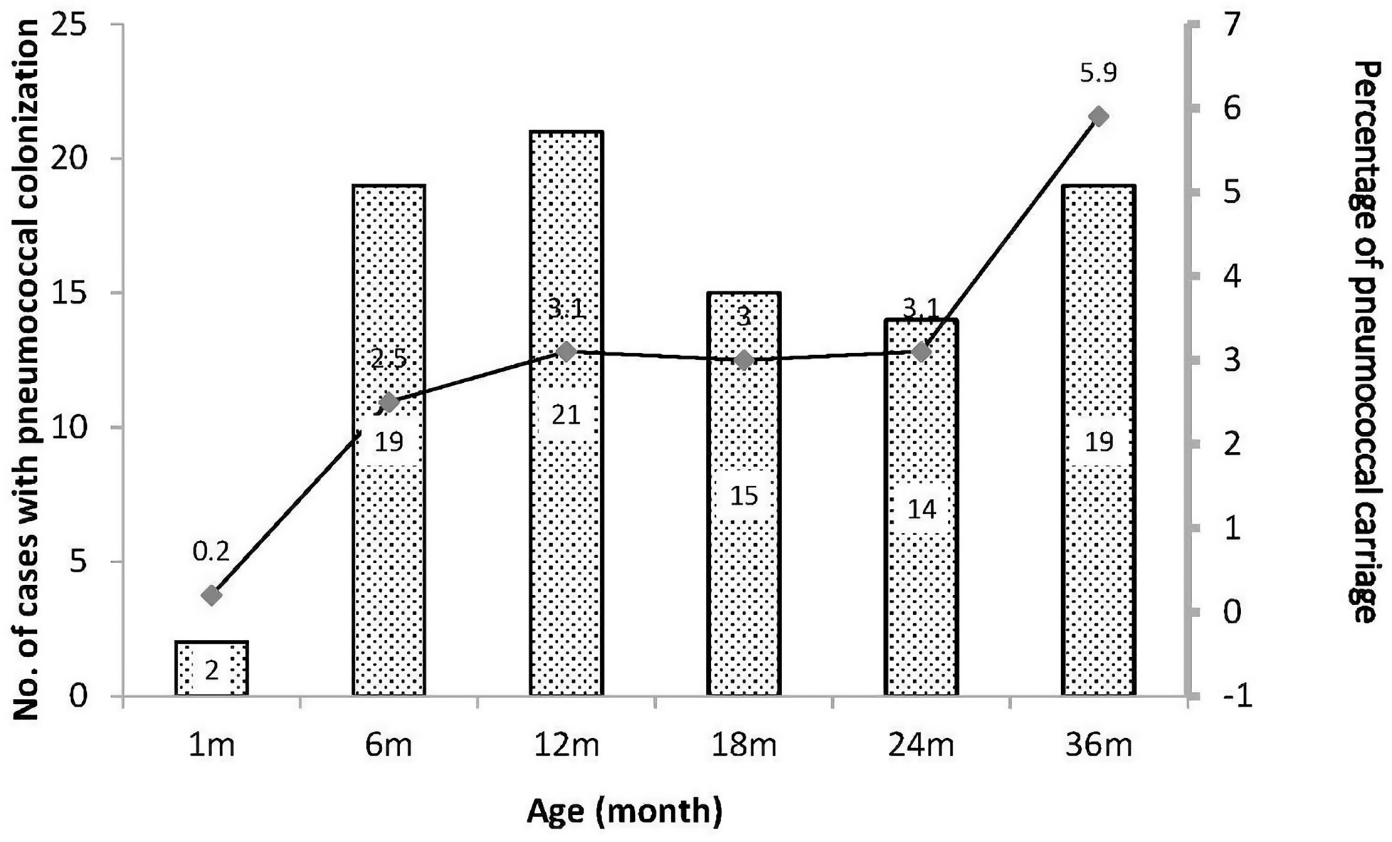
Trend of *Streptococcus pneumoniae* carriage at scheduled time points during the first three years of life.

From March 2013 to December 2017, 900 prenatal infants were recruited and 45 postnatal were excluded: 40, low birth weight (<2000 g); four, ventilator had been used after birth; and one, cerebral palsy. Hence, 855 infants were enrolled, with the numbers of collected samples in different age groups being 834 (1 month), 749 (6 months), 677 (12 months), 504 (18 months), 449 (24 months), and 323 (36 months). Nasopharyngeal culture was used for pathogen detection of all collected samples, and 107 pneumococcal isolates were recovered from 95 cases during the study period. Of the pneumococcal colonization time points during the first three years of infant life, pneumococcal colonization was found in 0.2% (2/834) at 1 month, followed by 2.5% (19/749) at 6 months, 3.1% (21/677) at 12 months, 3.0% (15/504) at 18 months, 3.1% (14/449) at 24 months, and 5.9% (19/323) at 36 months. Fig 1 shows the trend of *S*. *pneumoniae* carriage at the scheduled sampling time points during the first 36 months of infant life.

Of the 95 cases with pneumococcal colonization, 82 (86.3%) had received PCV13 and 8 (9.8%, 8/82), 31 (37.8%), 20 (24.4%), 23 (28.0%) of them had one, two, three and four doses of PCV13, respectively. Then we investigated factors associated with the occurrence of pneumococcal colonization and the result is shown in Table 1. For the selection of non-pneumococcal carriers as the control group, each pneumococcal carrier was frequency-matched with the non-carrier on variables of year of study, season, age and gender. Finally, 92 carriers and 172 non-carriers were chosen for the investigation. Three factors including demographic characters, environmental features and health conditions, were compared between pneumococcal carriers and non-pneumococcal carriers. Except day-care center attendance (11.6% versus 3.8%, *p* = 0.04), no other variable was found to be significantly associated with the occurrence of pneumococcal carriers. Furthermore, multiple logistic regression analysis also revealed some evidence that day-care center attendance was associated with pneumococcal carriage (OR 3.16; 95% CI 0.99–10.11; *p* = 0.05).

**Table 1:** Univariate and multivariate analysis of factors associated with pneumococcal colonization among the study population.

Factors associated with pneumococcal colonization among the study population by univariate logistic regression analysis				
Variables	Value		Odds ratio (95% confidence interval)	*p*-value
	Cases with pneumococcal colonization N = 92	Cases without pneumococcal colonization N = 172		
**Demographics**				
Male (%)	48/92 (52.2)	89/172 (51.7)	0.98 (0.59–1.63)	0.95
Age (mo)	17.6 ± 11.1	17.6 ± 11.1	1.00 (0.98–1.02)	0.97
Breast feeding*				
Breast feeding (%) since birth	82/92 (89.1)	157/172 (91.3)	1.28 (0.55–2.97)	0.57
Breast feeding period (mo)	6.7 ± 6.9	6.0 ± 6.1	1.02 (0.98–1.06)	0.38
Exclusive breastfeeding period (mo)	5.1 ± 6.8	3.6 ± 5.5	1.04 (1.00–1.09)	0.06
Pneumococcal vaccine				
Vaccinated infants (%)	81/92 (88.0)	146/172 (84.9)	1.31 (0.62–2.79)	0.48
Age of initial vaccination (mo)	3.2 ± 3.8	2.9 ± 3.3	1.00 (0.98–1.02)	0.97
No. of injected vaccine	2.4 ± 1.3	2.4 ± 1.4	0.98 (0.82–1.19)	0.86
**Environmental**				
Passive smoking (%)	46/90 (51.1)	73/170 (42.9)	1.39 (0.83–2.32)	0.21
No. of the family members	5.1 ± 1.9	4.2 ± 1.6	1.29 (0.94–1.78)	0.12
No. of children in the family	1.9 ± 0.4	1.6 ± 0.7	2.00 (0.54–7.44)	0.30
Day-care center attendance (%)	8/69 (11.6)	5/130 (3.8)	3.28 (1.03–10.44)	0.04
**Health conditions**				
PROM^†^ at birth (%)	4/92 (4.3)	7/172 (4.1)	1.07 (0.31–3.76)	0.91
Preterm birth (%)	12/92 (13.0)	30/172 (17.4)	0.71 (0.34–1.46)	0.35
Delivery via NSD (%)	55/92 (59.8)	106/172 (61.6%)	1.08 (0.64–1.81)	0.77
PICU^‡^ admission at birth (%)	17/92 (18.5)	39/172 (22.7)	0.77 (0.41–1.46)	0.43
Vitamin D deficiency^§^ (%)				
Maternal Vitamin D deficiency	30/40 (75.0)	52/68 (76.5)	1.08 (0.43–2.69)	0.86
Infantile Vitamin D deficiency	27/32 (84.4)	55/70 (78.6)	0.68 (0.22–2.06)	0.49
URI^¶^ within 2 weeks (%)	19/79 (24.1)	40/122 (23.3)	0.65 (0.34–1.23)	0.19
**Factors associated with pneumococcal colonization among the study population by multiple logistic regression analysis**				
**Variables**	**Pneumococcal carriers vs. non-pneumococcal carriers**			
	**Odds ratio (95% confidence interval)**			***p*-value**
Exclusive breastfeeding period (mo)	1.02 (0.97–1.08)			0.44
Day care center attendance (%)	3.16 (0.99–10.11)			0.05

*Infant with breast feeding means that infant had a history of breast feeding including exclusive breast feeding for at least 4 weeks

^†^ PROM indicates pre-labor rupture of membrane. It is defined as rupture of the membrane of the amniotic sac and chorion ≧1 hour before the onset of labor.

^‡^ PICU indicates pediatric intensive care unit.

^§^Vitamin D deficiency denotes serum 25-hydroxyvitamin D level <20 ng/mL

^¶^ URI indicates upper respiratory tract infection, including pharyngitis, croup, acute otitis media and acute sinusitis.

### Serotype distribution among the pneumococcal isolates during the study period

**Fig 2:**
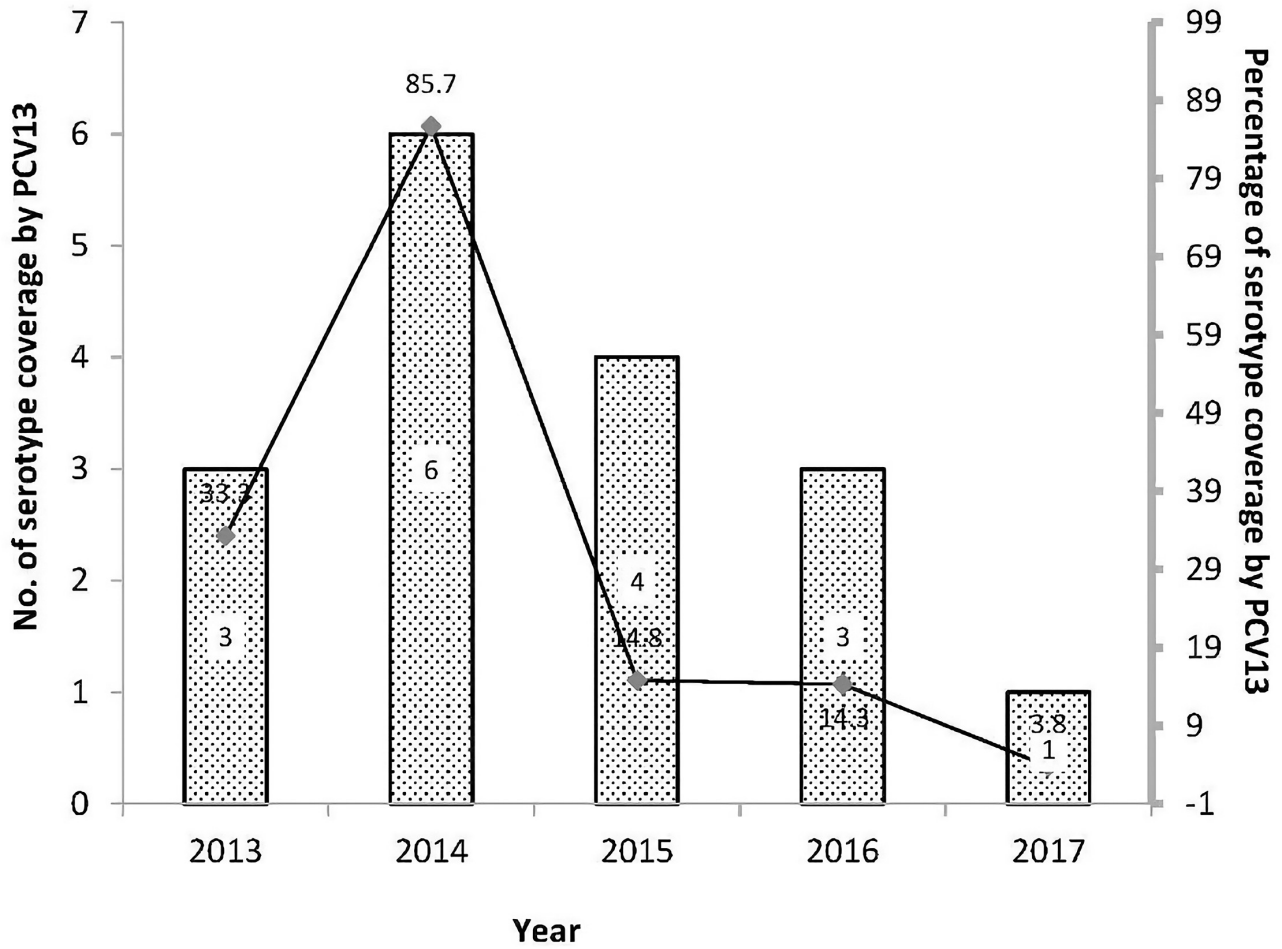
Trend of pneumococcal carriage isolates covered by the 13-valent pneumococcal conjugate vaccine during 2013–2017.

The distribution of serotypes of *S*. *pneumoniae* isolates is shown in Table 2. Of the total 107 isolates, 90 belonged to 13 different serotypes but the other 17 were non-typeable. In the 90 isolates with different serotypes, 81.1% (73/90) were non-PCV13 serotypes, with the most common serotype being 23A (33.3%, 30/90), followed by 15A (22.2%, 20/90), 15C (8.9%, 8/90), 15B (7.8%, 7/90), and 19A (7.8%, 7/90). We further analyzed the proportion of isolates with PCV13 coverage during 2013–2017 and showed that PCV13 serotype isolates were 33.3% (3/9) in 2013 and 85.7% (6/7) in 2014, respectively. However, a decrease occurred from 2014 to 2017: from 85.7% to 3.8% (1/26) and the decreasing trend during this period was statistically significant (*p* = 0.001) (Fig 2).

**Table 2:** Pneumococcal serotype distribution during the study period.

Year (N = 90)				2013 (9)	2014 (7)	2015 (27)	2016 (21)	2017 (26)
		Serotype	N (%)					
		6B	1 (1.1)			1 (3.7)		
		14	1 (1.1)	1 (11.1)				
	**PCV 7**	19F	3 (2.2)		2 (28.6)		1 (4.8)	
**PCV13**		23F	1 (1.1)			1 (3.7)		
		6A	4 (4.4)	2 (22.2)	1 (14.3)		1 (4.8)	
		19A	7 (7.8)		3 (42.9)	2 (7.4)	1 (4.8)	1 (3.9)
		15A	20 (22.2)	4 (44.4)	1 (14.3)	6 (22.2)	6 (28.6)	3 (11.5)
		15B	7 (7.8)			4 (14.8)		3 (11.5)
		15C	8 (8.9)	1 (11.1)		3 (11.1)	2 (9.5)	2 (7.7)
**Non-PCV13**		22F	1 (1.1)				1 (4.8)	
		23A	30 (33.3)	1 (11.1)		10 (37.0)	7 (33.3)	12 (46.2)
		34	5 (5.6)				1 (4.8)	4 (15.6)
		35B	2 (2.2)				1 (4.8)	1 (3.9)

Abbreviations: PCV, pneumococcal conjugate vaccine

### Antimicrobial susceptibility of pneumococcal isolates during the study period

Of the 90 pneumococcal isolates with identified serotypes, eight were unrecoverable and 82 were collected for antimicrobial susceptibility examination. Except 10 cases with antimicrobial agent prescription due to respiratory tract infection, most of the cases (87.8%, 72/82) did not receive antibiotic therapy within 2 weeks before sampling. For penicillin, the rates of I and R isolates were 76.8% (63/82) and 23.2% (19/82), respectively, and no S isolate was found. For ceftriaxone, the rates of S, I, and R isolates were 64.6% (53/82), 31.7% (26/82), and 3.7% (3/82), respectively. Table 3 shows the results for the penicillin susceptibility analysis of pneumococcal isolates during the study period.

**Table 3:** Penicillin susceptibility of colonized pneumococci during the study period.

Year (N = 82)		Serotype	2013 (9)		2014 (7)		2015 (24)		2016 (17)		2017 (25)	
**Antimicrobial susceptibility**			I	R	I	R	I	R	I	R	I	R
		6B					1					
		14		1								
	**PCV 7**	19F				2				1		
**PCV13**		23F					1					
		6A	2		1							
		19A				3		2		1	1	
		15A	1	3		1	4	2	6		3	
		15B					1	1			3	
		15C	1				3		1		2	
**Non-PCV13**		22F							1			
		23A	1				8	1	4	1	12	
		34									4	
			5(55.6)	4(44.4)	1(14.3)	6(**85.7**)	18(75.0)	6(**25.0**)	14(82.4)	3(**17.6**)	25(100.0)	

Abbreviations: I, intermediate; R, resistant; PCV, pneumococcal conjugate vaccine

We further found that rates of penicillin R isolates decreased from 85.7% (6/7), 25.0% (6/24), and 17.6% (3/17) to 0% during 2014–2017. Furthermore, the rates of I isolates increased from 14.3% (1/7), 75.0% (18/24), and 82.4% (14/17) to 100% during the same period. The decrease of penicillin R isolate rates during this period was statistically significant (*p*<0.001). Of the penicillin R isolates during 2014–2017, 60% (9/15) belonged to PCV13 serotypes (19A in 6 isolates and 19F in 3 isolates). However, most (91.4%, 53/58) of the penicillin I isolates were non-PCV13; 23A (45.3%, 24/53) was the most common serotype, followed by 15A (24.5%, 13/53), 15C (11.3%, 6/53), 15B (7.5%, 4/53), and 34 (7.5%, 4/53), respectively.

### Distribution of sequence types and serotypes during the study period

Eighteen different STs were identified during the study period, with 3 major STs being found in 63.3% (57/90) of the isolates, including ST 63 (30.0%, 27/90), ST 338 (21.1%, 19/90) and ST 166 (12.2%, 11/90). The largest clone, ST 63, included the majority of isolates from serotype 15A (74.1%, 20/27). All isolates from the second (ST 338) and third (ST 166) clones belonged to serotype 23A. Both serotype 15A and serotype 23A were non-PCV13 serotypes. Table 4 shows the distribution of sequence types and their association with serotypes during the study period.

**Table 4:** Distribution of sequence types and their association with serotypes during the study period.

Sequence type	Total			Year			PCV7/PCV10 serotypes				PCV13 serotype		Non-vaccine serotypes						
		2013	2014	2015	2016	2017	19F	6B	14	23F	19A	6A	15A	15B	15C	22F	23A	34	35B
	90	9	7	27	21	26	3	1	1	1	7	4	20	7	8	1	30	5	2
**Clone**																			
83	6	1		2	1	2								2	4				
2652	1	1							1										
63	27	4	1	10	7	5					1		20	2	4				
338	19	1		10	4	4											19		
81	3	1	1		1							3							
282	1	1										1							
320	6		3	2	1						6								
271	1		1				1												
236	2		1		1		2												
7479	1			1										1					
242	1			1						1									
95	1			1				1											
9395	5				1	4												5	
433	1				1											1			
166	11				3	8											11		
558	2				1	1													2
7502	1					1								1					
2889	1					1								1					

Abbreviations: PCV, pneumococcal conjugate vaccine

## Discussion

Our study showed that the prevalence of pneumococcal colonization gradually increased during the first three years of life, which is consistent with other studies [18,19]. Moreover, in our previous report, the prevalence of *Staphylococcus aureus* decreased in the same period, and an inverse relationship between the prevalence of *S*. *pneumoniae* and *S*. *aureus* was also reported by Labout, *et al*. [19,20]. This phenomenon might be due to pneumococcal competition, which is mediated via the production of hydrogen peroxide by *S*. *pneumoniae* or interference by *S*. *aureus* present in the nasopharyngeal spaces [21,22].

We also found that the overall prevalence of *S*. *pneumoniae* colonization was lower in our cohort than that has been reported in other studies [11,18]. One cause of this discrepancy may be due to different age groups between our study and others. Kuo, *et al*. reported that 14.1% of children had pneumococcal colonization and 57.6% were aged 2 to 5 years, the highest colonization age group of children less than 5 years [18]. However, in our series, most of them (65.3%, 62/95) were aged less than 2 years. Another cause may be because various PCV immunization programs had been implemented in Taiwan prior to our study. Of the statistical data reported by Wei, *et al*., a total of 128,680 and 114,140 doses of PCV10 were imported in 2010 and 2011, which would provide complete immunization for 20.5% and 15.2% of infants born in the corresponding years with the 3+1 schedule, respectively [23]. Wei, *et al*. also reported that 2,027,160 doses of PCV13 were imported during 2011–2013, which would provide complete immunization for 85.9% of infants born during the respective period using the 3+1 schedule [24]. We thought that these implementations prior to 2013 might have an impact on the pneumococcal colonization rate during the study period from 2013 to 2017. Besides, in our study, day-care center attendance was shown as a factor associated with the occurrence of pneumococcal carriage, which increased the risk of pneumococcal carriage by around threefold. It suggested that horizontal transmission among the pneumococcal colonized infants through the surrounding carriers, especially day-care center members, may be important for the acquisition of pneumococci in early childhood.

Serotype epidemiology of invasive pneumococcal disease (IPD) in Taiwan prior to 2013 revealed that unlike the decreasing trend of IPD caused by PCV7 serotypes, IPD caused by PCV13 serotypes decreased in the first years after PCV7 introduction in late 2005 and restored in 2008–2013 [24]. The discrepancy of trends of IPD caused by PCV7 and PCV13 serotypes was related to the emergence of IPD caused by the replacement serotypes not in PCV7 but was covered by PCV13, especially serotype 19A. Besides 19A (38.2%), PCV7 serotypes including 14 (11.6%), 23F (10.7%), 19F (10.1%) and 6B (8.3%), were the most common ones prior to 2013 [23]. Of our study, with respect to the serotype distribution during 2013–2017, the decrease of PCV13 serotypes occurred from 2014 to 2015, when the universal PCV13 vaccination program was initiated in Taiwan. In addition, 81.1% were non-PCV13 serotypes, with the most common ones being 23A, 15A, 15C, and 15B. We also found that the clonality of the leading non-PCV13 serotypes to be ST338^23A^ and ST63^15A^, and this result is consistent with the report by Su, *et al*. [2]. In the United States, ST 338^23A^ was observed in the pre-PCV7 era through capsular switch or structural change from the pre-existing ST338^23F^ strain [25]. In Taiwan, serotype 23F was the most common serotype before PCV13 introduction, despite limited information on the ST distribution [11]. Limited amount of ST338^23A^ distribution was documented during the same period [8,9]. All these findings suggested that similar to the findings in the US, the capsular switch from ST338^23F^ to ST338^23A^ may also have occurred in Taiwan, with increasing use of PCV13 resulting in an increase in ST338^23A^.

Regarding the association of PCV13 with antimicrobial susceptibility, we found that PCV13 administration might have reduced the rates of penicillin R isolates among pneumococcal isolates. Moreover, most of the penicillin R isolates belonged to PCV13 serotypes. Notably, Degan, *et al*. found that PCV7/PCV13 serotypes 6B, 9V, 14, 19F, and 23F and PCV13-specific serotypes 6A and 19A were the most common observed antibiotic-resistant serotypes [26]. Their study also showed that the immune responses against 6A, 19A, and 19F were significantly higher after PCV13 administration than those after PCV7 administration [27]. Thus, PCV13 had an added benefit over PCV7 in reducing the carriage of antibiotic-nonsusceptible *S*. *pneumoniae*.

There were some limitations in our study. First, it is an ongoing prospective study but the research period was limited from 2013 to 2017; thus, the initially enrolled cases could not all complete the first 36-month study. Second, the number of isolates was relatively small in our series. Based on these reasons, the selection bias might occur and thus the current result might be interpreted with caution. However, our study, a prospective cohort-designed study, may reduce the possibility of selection bias for the comparison because in a cohort study, the outcome is not known at case enrollment when exposure status is established. Nevertheless, selection bias can occur in retrospective studies since the outcomes have already occurred at the time of selection.

## Conclusions

Our study showed that under conditional PCV13 vaccination, the replacement of serotypes from PCV13 to non-PCV13 serotypes might be found along with the evolution of pneumococcal antibiotic susceptibility. The replacement non-PCV13 serotypes yielded lower rates of penicillin R isolates, suggesting that PCV13 may reduce the carriage of antibiotic-nonsusceptible *S*. *pneumoniae*, the burden of antibiotic-nonsusceptible pneumococcal diseases, and broad-spectrum antibiotic use.

## Abbreviations

PCV: pneumococcal conjugate vaccine
MIC: minimum inhibitory concentration
I: intermediate
R: resistant
PCR: polymerase chain reaction
MLST: multi-locus sequence typing
ST: sequence type
